# 

**Published:** 1881-01

**Authors:** 


**TERMS.—50 Cents a Year. Address The Bistoury, Elmira, N. Y.**

### SUBSCRIPTIONS RECEIVED TO JANUARY, 1881.

NEW YORK—Mrs P A LaFrance, Thomas M Howell, Mrs T W Crane $2, Dr Wm W Greene, Geo W Waters, Henry A Best $1, Dr Geo F Hand, Dr R L Van Kleek, C J Langdon $2, M C Fuller $2, Dr W H Gilbert, Dr. N R Stanford $2, Dr Jas Hildebrand $2, Dr G H Dudley $3, Hiram D Voe $4, Simon H Hilfish $2, Dr H H Smith $2, Dr Moyer & Son $2, Drs French & Underhill $3, Dr D D Hines $1, Dr L Dart $1, Dr F D Morse $1, Dr J Eastabrook $1, Dr Coryell $1, Dr Dan'l Smith $1, Dr D Brown $2, Dr Myron Samuels $2, Mrs D H Ford $1, M H Dodd $2, L B Lawrence $1, F Howe $1, Dr A B George $1, O J Beardsley 1, Saml H Doxcy $1, David Smith $1, D D Crouse, Mrs Jas Highland $1, L P Miner, J D Lyttle, Dr H French $1, Mrs J D Paine, F W Sanders, Dr Davidson, Dr P H Corson $2.

PENNSYLVANIA—Dr. T D Gray, J H Shearer $1, Dr Wm M Sanders $2, Dr Jas H Leisenring $4, Dr Fayette Crouse $2, Dr D D Leslie $1, Dr N House $2, Dr Lamott Smith $2, Dr G H Dimon $1, Dr Hottenstein $2, Dr R Shaffer $2, Dr D Snyder $1, Dr T White $1, Dr A Osbourne $2, Dr Wesley Brown $2, Mrs F A Hines $1, J H Hollister $1, Max Henderson $2, L B Fullner $1, Dr A N Hillbish $2, Dr O Parks $1, Dr. J B Freeman $2, Dr Sam Garret $1.

ARKANSAS—Dr N L Messengale, Mrs J Blood, Dr Jas Frothingham $2, Dr W G Wright, Jos P Angell.

ALABAMA—Dr Ike Engleson, Dr P B Hampton.

COLORADO—Dr D B James $6.

CONNECTICUT—Dr T D Snow, Mrs F Henderson.

FLORIDA—Dr Furgeson $1, Dr A A Lovejoy $2

IOWA—S P Kennedy $1.

INDIANA—Dr Harvey Smith $2, Dr T G Holmes $1, Dr Frank English $1, Dr A D Simonson.

ILLINOIS—Geo M Creed, Mrs D Lamont $1, Dr H H Squires $1, Dr O B Howe $1.

KENTUCKY—John Banks $1. Dr S S Freelingheisen $2, Dr Dan Emmett $1.

MISSOURI—Rev H Marks, Dr L P Dodge, Dr Frank Fields, Dr J P Helmbold $2.

OHIO—Hon J T Up de Graff.

TEXAS—Dr L H Kitchel.

